# A systematic review and meta-analyses of glucagon-like peptide-1 receptor agonists in acute myocardial infarction

**DOI:** 10.1186/s43044-026-00767-y

**Published:** 2026-07-21

**Authors:** Jasmin Kaur Bhullar, Amol Bahekar

**Affiliations:** 1 Department of Internal Medicine, University of North Carolina Health Southeastern, Lumberton, USA; 2 Division of Interventional Cardiology, Cape Fear Valley Medical Center, Fayetteville, USA

**Keywords:** Glucagon-like peptide-1 (GLP-1) receptor agonists, Myocardial infarction, Acute coronary syndrome, Percutaneous coronary intervention (PCI), Myocardial infarction scar size, Heart failure

## Abstract

**Background:**

Previous research has shown that glucagon-like peptide-1 receptor agonists (GLP-1 RAs) have cardioprotective effects. However, their efficacy in acute myocardial infarction (AMI) is not yet well established. We conducted a systematic review and meta-analyses to assess the impact of GLP-1 RAs in AMI.

**Methods:**

We systematically searched major databases for studies in patients with ST-elevation (STEMI) or non-ST-elevation myocardial infarction (NSTEMI). These included MEDLINE (accessed via PubMed), Excerpta Medica dataBASE (Embase), Cochrane Central Register of Controlled Trials (CENTRAL), ClinicalTrials.gov, and Google Scholar from their inception through February 2026. PRISMA guidelines were used to conduct this review. Both randomized controlled trials (RCTs) and observational studies were included in the systematic review. However, only RCTs were included in the meta-analyses. The primary outcome was infarct size relative to the area at risk (AAR). Secondary outcomes included major adverse cardiovascular events (MACE) and safety outcomes (nausea, hypoglycemia, pancreatitis). The Cochrane Risk of Bias 2 (RoB 2) tool and Newcastle-Ottawa Scale (NOS) were used to assess risk of bias.

**Results:**

We included eight studies with a total of 1,483 participants. GLP-1 RAs led to a smaller infarct size indexed to the AAR compared with placebo. The mean reduction was 10.14% points (95% confidence interval [CI]: −14.11–−6.17; *P* < 0.001). The primary infarct-size analysis was based on only three RCTs. GLP-1 RAs led to an improvement in the left ventricular ejection fraction (LVEF). The absolute mean improvement was 2.93% points (95% CI: 0.18–5.67; *P* < 0.05). Subgroup analyses were conducted to explore the findings in greater detail. They revealed that liraglutide was associated with an even higher absolute increase in LVEF. The absolute mean improvement was 5.32% points (95% CI: 3.49–7.15; *P* < 0.01). Nausea was more frequent in the treatment group, while hypoglycemia and pancreatitis were not significantly increased. Limitations included relatively small sample sizes in the individual studies, short follow-up periods, and variability in study designs.

**Conclusions:**

In patients with AMI, GLP-1 RAs reduce relative infarct size and improve LVEF. No clear safety signal was detected in the available short-term trial data, except for increased nausea. These results indicate that GLP-1 RAs could be beneficial as an additional treatment in AMI.

**Supplementary Information:**

The online version contains supplementary material available at 10.1186/s43044-026-00767-y.

## Background

Despite major advances in acute care and reperfusion techniques, acute myocardial infarction (AMI) continues to be a major contributor to morbidity [1]. Infarct size plays a central role in mortality and cardiovascular outcomes after an AMI [2]. Interventions that reduce myocardial injury may reduce infarct size and prevent adverse cardiac remodelling [3].

Glucagon-like peptide-1 (GLP-1) receptor agonists (GLP-1 RAs) are commonly prescribed in type 2 diabetes. Both preclinical and early clinical research showed that they confer additional cardioprotection beyond blood glucose reduction alone. This includes attenuation of myocardial injury, reduction of inflammation, and enhanced cardiac function [4–15]. In rodent models of ischemia–reperfusion injury, GLP-1 RAs reduced the infarct size and improved the cardiac function after the ischemic event [16, 17]. The GLP-1 RA lixisenatide reduced the infarct-size/area at risk ratio by 36% in rat hearts [17]. In-vitro studies suggest that the GLP-1 mediated cardioprotection is driven mechanistically by activation of the glucagon-like peptide-1 receptor (GLP-1R) and phosphatidylinositol-3 kinase (PI3K)-dependent pathway [18]. These results have contributed to growing interest in GLP-1 RAs as a potential additional therapy in AMI.

A number of clinical studies, including randomized controlled trials (RCTs), have assessed how GLP-1 RAs influence infarct size and cardiovascular outcomes in individuals with ST-elevation myocardial infarction (STEMI) and non-ST-elevation myocardial infarction (NSTEMI) [6–15]. However, these studies were frequently constrained by small cohort sizes, which makes it difficult to reliably interpret the results. Previous reviews and meta-analyses on this have been published before [19]. The most recent review provided only a qualitative analysis, without performing a quantitative synthesis [19]. Earlier meta-analyses did not incorporate all currently available RCTs on this topic [20–22].

Against this background, we performed a systematic review of clinical studies and a meta-analyses of RCTs to quantify the impact of GLP-1 RAs on infarct size relative to the area at risk (AAR), as well as on left ventricular ejection fraction, major adverse cardiovascular events, relevant biomarkers, and safety outcomes in AMI. This study aimed to examine whether GLP-1 RAs would be a beneficial additional medication in AMI for future research and practice.

## Methods

We adhered to the Preferred Reporting Items for Systematic Reviews and Meta-Analyses (PRISMA) guidelines in this study [23] (see Supplementary Tables S4 and S5). No protocol was registered prior to conducting this systematic review and meta-analyses.

### Search strategy

A systematic and comprehensive literature search was conducted to identify all relevant studies. We queried multiple databases (MEDLINE (accessed via PubMed), Excerpta Medica dataBASE (Embase), Cochrane Central Register of Controlled Trials (CENTRAL), ClinicalTrials.gov, and Google Scholar) from their inception through February 2026. The search focused on comparative studies assessing GLP-1 RAs in AMI. The search keywords included glucagon-like peptide-1 receptor agonists, exenatide, liraglutide, semaglutide, albiglutide, taspoglutide, dulaglutide, lixisenatide, efpeglenatide, and myocardial infarction. Boolean logic (“AND”, “OR”) was applied to combine these keywords. The ClinicalTrials.gov trial registry was also searched for unpublished and ongoing studies. The complete search strategy is outlined in the supplementary data (see Supplementary Table S1). No additional filters or language restrictions were used. In addition, reference lists of included articles were manually screened to ensure inclusion of all relevant studies.

### Study selection

We restricted the inclusion to studies that were either randomized trials or observational analyses with a comparator arm. Only studies with patients presenting with an AMI, encompassing both ST-elevation myocardial infarction (STEMI) or non-ST-elevation myocardial infarction (NSTEMI), were included. Eligible interventions involved treatment with any kind of GLP-1 RA. The comparator group consisted of patients receiving placebo or standard therapy. Studies without a comparator group were excluded. Both randomized controlled trials (RCTs) and observational studies were included in the systematic review. However, only RCTs were included in the meta-analyses. Study selection was performed independently by two reviewers. The study selection began with screening of the titles and abstracts and was followed by full-text assessment. Discrepancies were resolved by consensus discussion. When multiple reports were identified from overlapping patient cohorts, all reports were considered for inclusion. For outcome-specific meta-analyses in which only one report provided data for a given outcome, that report was used. When multiple reports from an overlapping cohort reported the same outcome, only the report with the largest sample size for that outcome was included to avoid double-counting of patients.

### Outcome measures

Our study focused on relevant imaging, clinical, and laboratory outcomes after the administration of GLP-1 RAs in AMI. The primary outcome was infarct size, expressed as a proportion of the area at risk (AAR) (infarct size/AAR, expressed as a percentage). For each study, the absolute mean differences between treatment and control groups were calculated in percentage points and used as the summary effect measure for pooled analysis. Secondary outcomes included major adverse cardiovascular events (MACE) as defined in the individual studies. Our secondary safety outcomes were nausea, hypoglycemia, and pancreatitis.

We extracted information on authorship, year of publication, country, sample size, study design, intervention characteristics, patient demographics (age, sex), comorbidities (diabetes, hypertension, hyperlipidemia, and smoking status), and body mass index (BMI). The extraction and review of data were carried out independently by two investigators. Disagreements were resolved through discussion and mutual agreement. If information was incomplete, we contacted the corresponding authors for clarification. No estimations or imputations of the original data were undertaken.

### Quality assessment

We assessed the risk of bias in the included RCTs by using the Cochrane Risk of Bias 2 (RoB 2) tool. Each study was analyzed across a fixed set of five domains of bias. These domains were: randomization, deviations from intended interventions, missing data, outcome measurement, and selection of the reported result. Based on these criteria of the RoB2 tool, studies were categorized as having low risk of bias, high risk of bias, or some concerns regarding risk of bias [24]. A risk-of-bias plot based on the RoB2 assessment was generated using the robvis tool [25]. We assessed the quality of the included retrospective study by using the Newcastle-Ottawa Scale (NOS) [26]. Two investigators independently examined the quality of the included studies. Disagreements among the reviewers were resolved by consensus.

### Statistical methods

All statistical analyses were carried out by using the software R (version 4.5.2; R Foundation for Statistical Computing, Vienna, Austria) and RStudio (version 2026.1.1.403; Posit Software, Boston, MA, USA) with the packages rstudioapi (version 0.19.0), readxl (version 1.5.0), and meta (version 8.5-0). A random-effects model was used for all pooled analyses. Mean differences (MD) with corresponding 95% confidence intervals (CI) were calculated for outcomes measured on a continuous scale (infarct size in relation to AAR). Mean differences were reported as absolute differences in percentage points between groups. Risk ratios (RR) with 95% confidence intervals were calculated as effect estimates for binary outcomes (MACE, nausea, and hypoglycemia). Double-zero event studies (i.e. studies in which no events occurred in either the intervention or control arm) for binary outcomes were displayed in the forest plot. However, they did not contribute to the estimation of relative treatment effects and therefore received zero statistical weight in the meta-analyses. Otherwise, no zero-event studies were identified for any outcome. Therefore, no continuity corrections or alternative zero-event handling methods were applied.

We quantified statistical heterogeneity by reporting the I² values. Heterogeneity was considered to be low when the I² value was below 25%, moderate when it ranged from 25% to 75%, and high when it exceeded 75% [27]. Chi-square (Q) test and tau-squared (τ²) values were also reported as additional measures of heterogeneity. Leave-one-out sensitivity analyses and subgroup analyses were carried out, where appropriate, to investigate possible causes of heterogeneity.

Assessment of publication bias and meta-regression analyses were not undertaken, as the number of included studies in the meta-analyses was less than 10.

## Results

### Systematic literature search, study characteristics and risk of bias assessment

#### Flow diagram of the study search and selection process

**Fig. 1:**
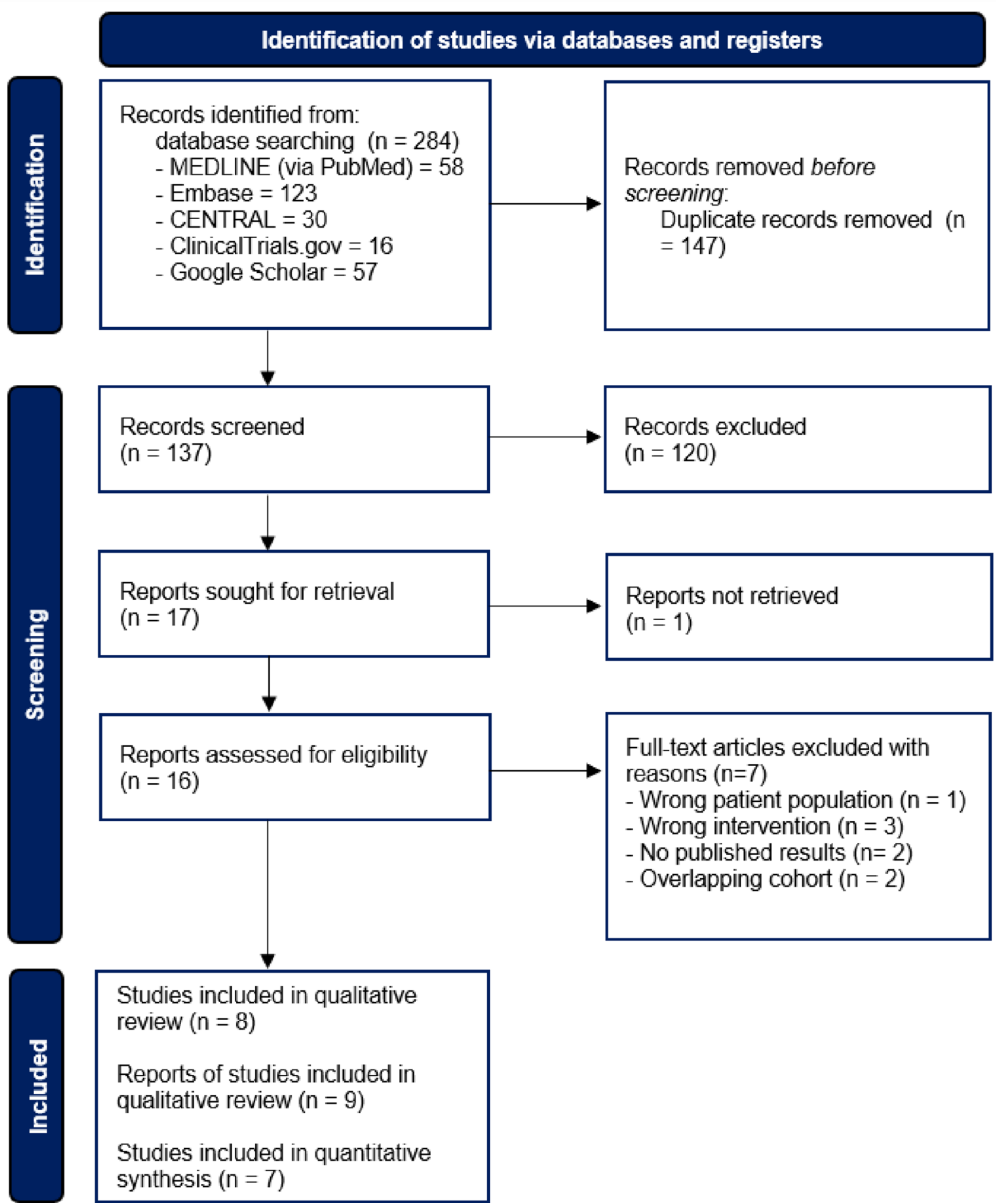
Flow diagram of systematic review and meta-analyses

We identified 284 potentially relevant publications in total through the database searches and reference list screening. The flow diagram for this selection process, which followed PRISMA guidelines, is shown in Fig. 1. Seven RCTs and one observational study met the predefined eligibility criteria and were ultimately included in the systematic review [6–8, 10–15]. Two reports (Bernink et al. [7] and Roos et al. [8]) of RCTs had overlapping patient cohorts, as Bernink et al. [7] was a pilot study for the subsequent trial reported by Roos et al. [8]. Because Roos et al. [8] included a larger number of patients, it was designated as the primary report and used for participant counts and all shared outcomes. Bernink et al. [7] was used exclusively for the meta-analysis of infarct size relative to the area at risk (AAR), as this outcome was not reported by Roos et al. [8]. The meta-analyses were based on eight reports from seven RCTs.

#### Characteristics of included studies

An overview of the baseline characteristics for the included studies is provided in Tables 1 and 2. The study sample sizes ranged from 15 to 387. Eight studies, which were published between 2012 and 2021, were included in the systematic review. They encompassed a total of 1,483 participants. Seven RCTs were included in the meta-analyses. The included RCTs enrolled a total of 1,468 participants.

Six of the included RCTs focused on patients with STEMI, one RCT on patients with NSTEMI, and one retrospective study included both. Regarding GLP-1 RA administration, exenatide was administered in four studies and liraglutide in four studies. One of the RCTs was a 2 × 2 factorial trial. For this factorial trial, we used the published effect estimates for exenatide versus no exenatide, since separate data isolating exenatide without concomitant interventions were not available [14]. The only observational study that could be found in addition to the seven RCTs was a single retrospective study with liraglutide. It only included a small sample size of 15 patients. Its retrospective study design was inconsistent with the other studies, which were RCTs. It also had a serious risk of bias [15]. Accordingly, the meta-analyses are based solely on seven RCTs.

Three of the included RCTs provided precise numerical data for the primary outcome [6, 7, 12]. Major adverse cardiovascular events were defined in the individual studies as various combinations of the outcomes MI (myocardial infarction), cardiac death, stent thrombosis, repeated revascularization, and stroke. The majority of the included studies quantified side effects such as nausea, hypoglycemia, and pancreatitis.

**Table 1:** General study characteristics

	Intervention	Comparator	Study design	Country	Study duration	Follow-up	AMI type	Imaging modality	Reported outcomes
Lønborg, 2012 [6] (*N* = 387)	IV Exenatide at 0.12 µg/min for 15 min (min) before procedure, then maintained at 0.043 µg/min for 6 h (*n* = 196)	Placebo (*n* = 191)	RCT	Denmark	2009–2015	3 months	STEMI	CMR	Infarct size relative to AAR, LVEF, hypoglycemia, MACE
Bernink, 2013 [7] (*N* = 71)	IV Exenatide 5 µg before procedure for 30 min, then continuous infusion of 20 µg/24 h for 72 h (*n* = 35–36)	Placebo (*n* = 35–36)	RCT	The Netherlands	2009–2012	4 months	STEMI	CMR	Infarct size relative to AAR
Woo, 2013 [10] (*N* = 116)	SC 10 µg and 10 µg IV bolus of Exenatide 5 min before reperfusion. SC 10 µg twice daily on following 2 days (*n* = 39)	Placebo (*n* = 77)	RCT	Korea	2009–2011	6 months	STEMI	Echocardiography and CMR	LVEF, nausea, hypoglycemia
Chen, 2016a [11] (*N* = 210)	SC Liraglutide 1.8 mg 30 min before PCI (*n* = 105)	Placebo (*n* = 105)	RCT	China	2015–2016	3 months	STEMI	Echocardiography	LVEF
Chen, 2016b [12] (*N* = 96)	SC Liraglutide 1.8 mg 30 min before PCI, 0.6 mg for 2 days, 1.2 mg for 2 days, followed by 1.8 mg for 3 days (*n* = 49)	Placebo (*n* = 47)	RCT	China	2013–2016	6 months	STEMI	Echocardiography and CMR	Infarct size relative to AAR, LVEF, nausea, hypoglycemia, pancreatitis, MACE
Chen, 2016c [13] (*N* = 90)	SC Liraglutide 0.6 mg for 2 days, 1.2 mg for 2 days, followed by 1.8 mg for 3 days (*n* = 45)	Placebo (*n* = 45)	RCT	China	2015–2016	6 months	NSTEMI	Echocardiography	LVEF, nausea, hypoglycemia, pancreatitis, MACE
Nozue, 2016 [15] (*N* = 15)	Retrospective analysis of diabetic patients on liraglutide – did not specify a particular treatment dosage (*n* = 6)	Standard therapy (*n* = 9)	Retrospective cohort	Japan	2011–2015	6 months	STEMI and NSTEMI	CMR	LVEF
Roos, 2016 [8] (*N* = 191) (also included patients from a previous pilot study from Bernink et al. in 2013 [7])	IV Exenatide 5 µg before procedure for 30 min, then continuous infusion of 20 µg/24 h for 72 h (*n* = 96)	Placebo (*n* = 95)	RCT	The Netherlands	2009–2016	4 months	STEMI	CMR	Nausea, hypoglycemia, MACE
García Del Blanco, 2021 [14] (*N* = 378)^†^	IV Exenatide at 0.12 µg/min for 15 min before procedure, then maintained at 0.043 µg/min for 6 h (*n* = 189)	Placebo (*n* = 189)	RCT	Spain	2016–2020	1 week	STEMI	CMR	LVEF

AMI: acute myocardial infarction; IV: intravenous; RCT: randomized controlled trial; STEMI: ST-elevation myocardial infarction; CMR: cardiovascular magnetic resonance; AAR: area at risk; LVEF: left ventricular ejection fraction; MACE: major adverse cardiovascular events; SC: subcutaneous; PCI: percutaneous coronary intervention; NSTEMI: non-ST-elevation myocardial infarction

**Table 2:** Baseline characteristics of the participants in the included studies

	Age, years	Male, *n* (%)	Diabetes mellitus, *n* (%)	Hypertension, *n* (%)	Hyperlipidemia, *n* (%)	Smoking, *n* (%)	BMI (kg/m2)
Lønborg, 2012 [6] (*N* = 387)	Intervention: 62 ± 11 Control: 62 ± 12	Intervention: 160 (82) Control: 146 (76)	Intervention: 14 (7) Control: 21 (11)	Intervention: 73 (37) Control: 74 (39)	Intervention: 14 (7) Control: 17 (9)	Intervention: 49 (25) Control: 52 (27)	Intervention: 27 ± 4 Control: 28 ± 4
Bernink, 2013 [7] (*N* = 71)^†^	Intervention: 60 ± 10 Control: 58 ± 8	Intervention: 16 (84) Control: 15 (75)	Intervention: 0 Control: 0	Intervention: 8 (42) Control: 6 (30)	Intervention: 5 (26) Control: 6 (30)	Intervention: 12 (63) Control: 11 (55)	Intervention: 27 ± 4 Control: 27 ± 4
Woo, 2013 [10] (*N* = 116)	Intervention: 61.2 ± 10.8 Control: 59.1 ± 11.5	Intervention: 30 (77) Control: 63 (82)	Intervention: 10 (26) Control: 22 (28)	Intervention: 22 (56) Control: 43 (56)	Intervention: 12 (31) Control: 18 (23)	Intervention: 24 (61) Control: 37 (48)	Intervention: 24.9 ± 3.0 Control: 25.3 ± 3.1
Chen, 2016a [11] (*N* = 210)	Intervention: 58.2 ± 11.5 Control: 57.4 ± 11.3	Intervention: 71 (68) Control: 67 (64)	Intervention: 17 (16) Control: 21 (20)	Intervention: 59 (56) Control: 55 (52)	Intervention: 23 (22) Control: 20 (19)	Intervention: 68 (65) Control: 65 (62)	Intervention: 25.6 ± 3.3 Control: 25.3 ± 3.2
Chen, 2016b [12] (*N* = 96)	Intervention: 57.1 ± 13.0 Control: 58.7 ± 12.7	Intervention: 27 (69) Control: 26 (68)	Intervention: 5 (13) Control: 7 (18)	Intervention: 18 (46) Control: 19 (48)	Intervention: 4 (10) Control: 5 (13)	Intervention: 13 (33) Control: 14 (37)	Intervention: 25.2 ± 3.4 Control: 25.4 ± 3.2
Chen, 2016c [13] (*N* = 90)	Intervention: 58.02 ± 11.7 Control: 59.0 ± 12.1	Intervention: 34 (76) Control: 32 (71)	Intervention: 9 (20) Control: 13 (28)	Intervention: 27 (60) Control: 29 (64)	Intervention: 11 (24) Control: 9 (20)	Intervention: 25 (56) Control: 27 (60)	Intervention: 25.2 ± 3.4 Control: 25.0 ± 3.1
Nozue, 2016 [15] (*N* = 15)	Intervention: 68 ± 10 Control: 69 ± 9	Intervention: 6 (100) Control: 8 (89)	Intervention: 6 (100) Control: 9 (100)	Intervention: 3 (50) Control: 5 (56)	Intervention: NR Control: NR	Intervention: 3 (50) Control: 6 (67)	Intervetion: 24.6 ± 2.1 Control: 24.0 ± 4.1
Roos, 2016 [8] (*N* = 191)^†^ (also included patients from a previous pilot study from Bernink et al. in 2013 [7])	Intervention: 57.23 ± 10.2 Control: 57.48 ± 10.1	Intervention: 33 (79) Control: 36 (73)	Intervention: 0 Control: 0	Intervention: 9 (22.5) Control: 6 (13.3)	Intervention: 9 (23.1) Control: 12 (27.9)	Intervention: 18 (45) Control: 26 (55.3)	Intervention: 27.47 ± 4.1 Control: 26.39 ± 3.2
García Del Blanco, 2021 [14] (*N* = 378)^†^	Intervention: 61.7 ± 11.5 Control: 61.2 ± 11	Intervention: 88 (80) Control: 98 (87.5)	Intervention: 22 (20) Control: 25 (22.3)	Intervention: 45 (40.9) Control: 58 (51.8)	Intervention: 60 (54.5) Control: 67 (59.8)	Intervention: 42 (38.2) Control: 60 (53.6)	Intervention: 27.6 ± 3.9 Control: 27.1 ± 4

^†^ If intention-to-treat baseline characteristics were not reported, per-protocol data were utilized

NR: not reported; BMI: body mass index

#### Risk of bias assessment

Plots and a table in the supplementary data present the results of the risk of bias assessment obtained by two independent reviewers (see Supplementary Figures S1 and S2 and Supplementary Table S2). The Cochrane Risk of Bias 2 (RoB 2) tool [24] was used for this assessment of RCTs. Overall, the evaluation showed that the majority of the included RCTs were at low risk of bias. The results of this evaluation are displayed as a traffic light plot and a summary plot in the supplementary data (see Supplementary Figures S1 and S2). The risk of bias of the included retrospective study was assessed using the Newcastle-Ottawa Scale (NOS) [26]. It had a low risk of bias (see Supplementary Table S2).

### Primary outcome: myocardial infarction size in grams in relation to the area at risk (AAR) in grams

**Fig. 2:**
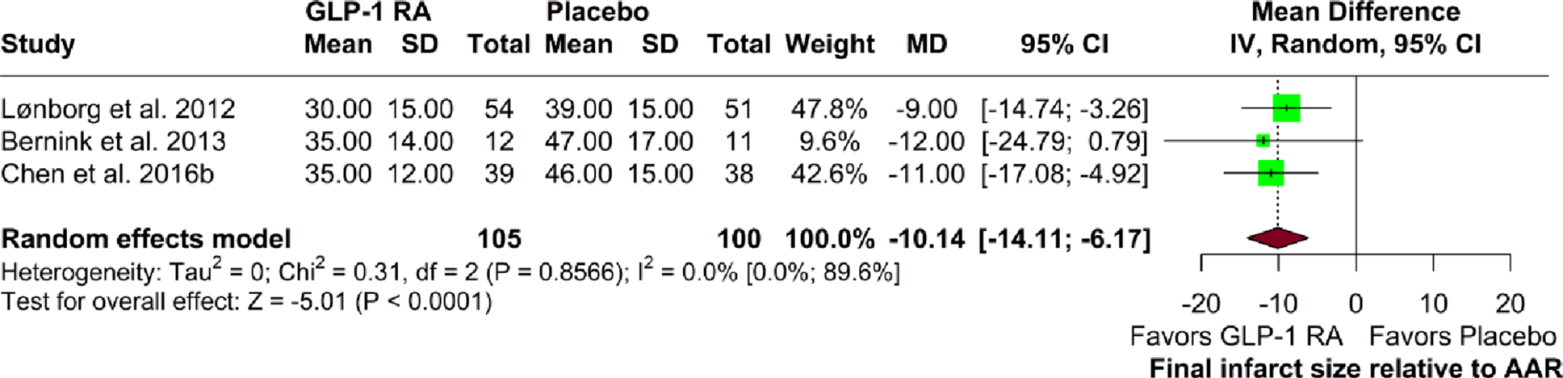
Forest plot comparing the primary outcome of final infarct size (in grams) relative to the area at risk (AAR) (in grams)

The primary outcome was reported as myocardial infarction size in grams in relation to the area at risk (AAR) in grams. The mean differences were calculated as the absolute difference in percentage points between groups. The results of the pooled analysis were calculated by using a random-effects model. They demonstrated that, in patients with AMI, GLP-1 RAs significantly reduced the myocardial infarction size (in grams) in relation to the area at risk (AAR, in grams) compared with the control group. The absolute mean reduction was 10.14% points (mean difference [MD] = -10.14; 95% confidence interval [CI]: -14.11– -6.17; *P* < 0.0001) [6, 7, 12]. The studies included in this pooled analysis demonstrated no statistical heterogeneity (I² = 0%, heterogeneity *P* = 0.86; see Fig. 2).

### Secondary outcomes

#### Left ventricular ejection fraction as a significant secondary outcome

**Fig. 3:**
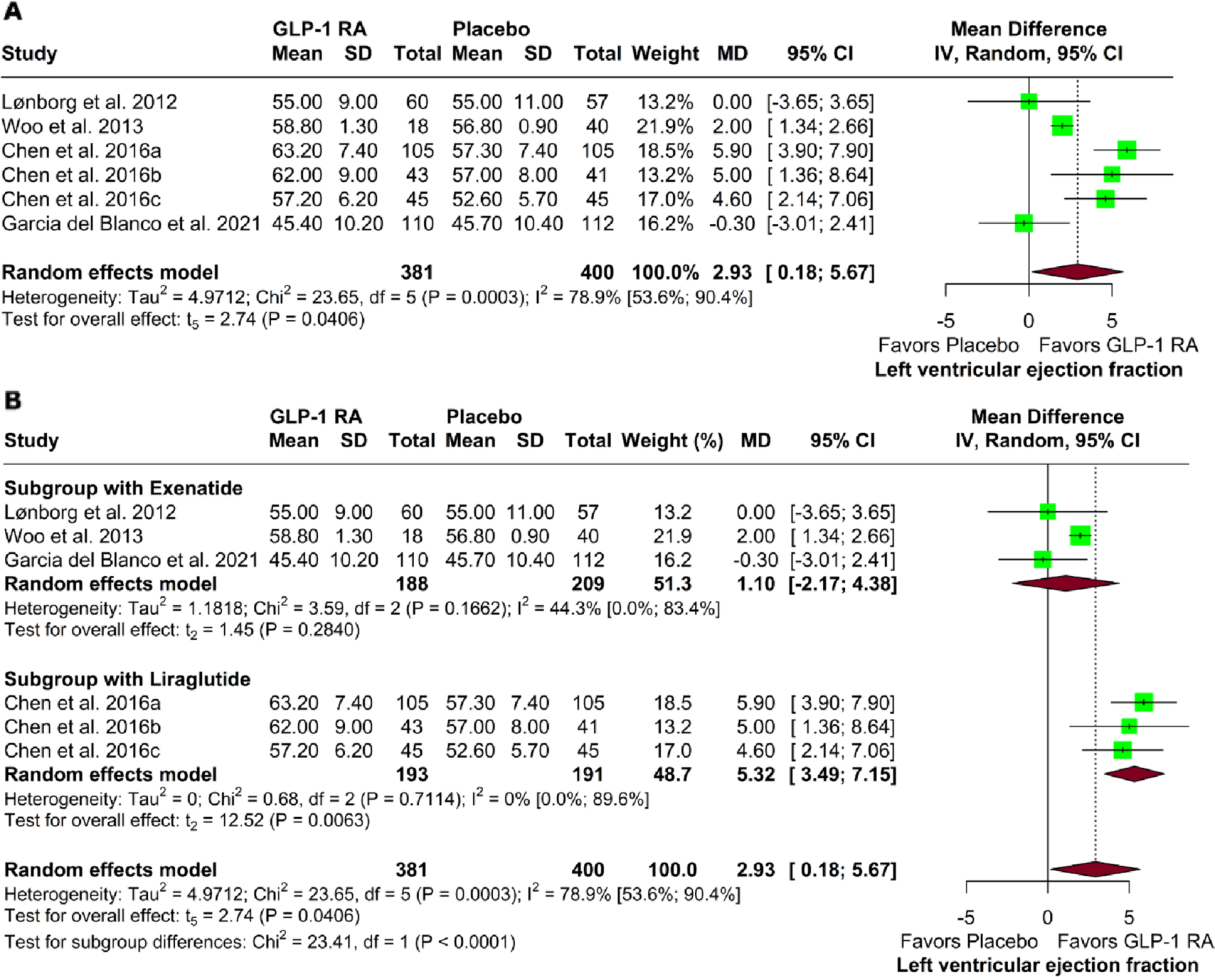
Secondary outcome of left ventricular ejection fraction (LVEF). **A** Forest plot comparing the LVEF. **B** Subgroup analysis comparing exenatide and liraglutide for LVEF

A pooled analysis of six RCTs showed that GLP-1 RAs significantly increased LVEF compared with control in patients with AMI. The absolute mean difference compared with the control group was 2.93% points (mean difference [MD] = 2.93; 95% CI: 0.18–5.67; *P* = 0.04; see Fig. 3A) [6, 10–14]. However, substantial heterogeneity was present across the studies (I² = 75.4% heterogeneity; *P* < 0.001). Subsequently, a subgroup analysis was performed. It is shown in Fig. 3B. It revealed significant subgroup differences between various GLP-1 RA drugs (*P* < 0.001). No significant improvement in LVEF was observed with exenatide. In contrast to this, liraglutide significantly increased the LVEF by 5.32% points compared with control (MD = 5.32; 95% CI: 3.49–7.15; *P* < 0.01; see Fig. 3B). Notably, no statistical heterogeneity was present among the liraglutide studies themselves. In line with these findings, the included retrospective study of patients with AMI likewise reported a trend toward higher LVEF in the liraglutide group.

An additional sensitivity analysis for this secondary outcome is presented in the supplementary data (see Supplementary Figure S4).

#### Other secondary outcomes

**Fig. 4:**
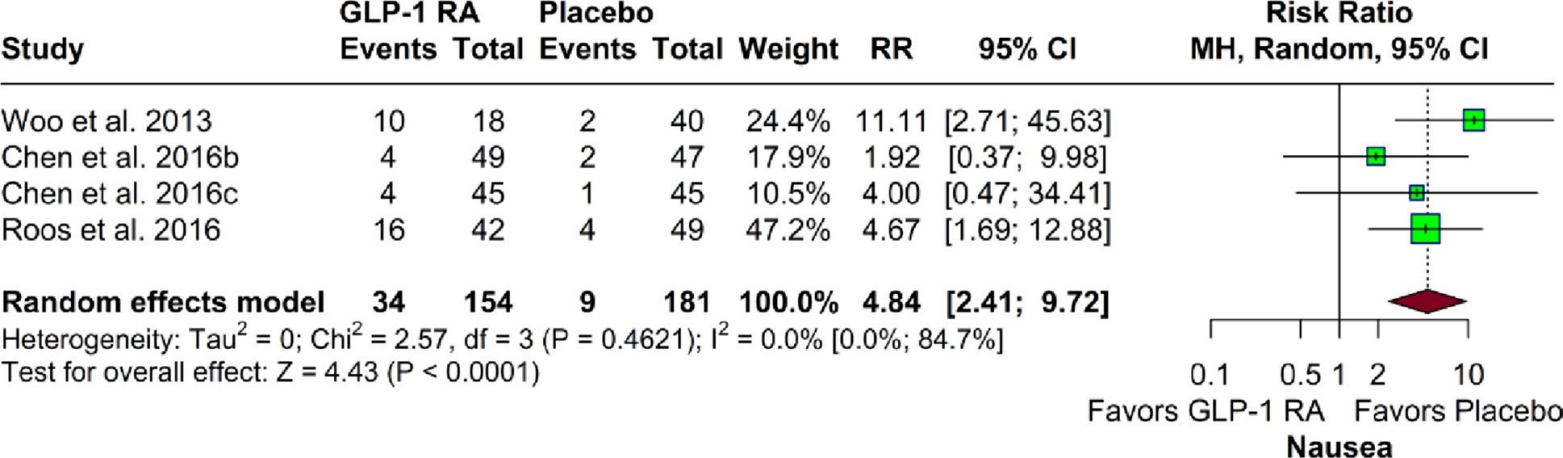
Forest plot of nausea

Among patients with AMI, GLP-1 RAs significantly increased the risk of nausea (RR = 4.84; 95% CI: 2.41–9.72; *P* < 0.01; see Fig. 4). However, in many cases the occurrence of nausea did not result in study discontinuation.

**Fig. 5:**
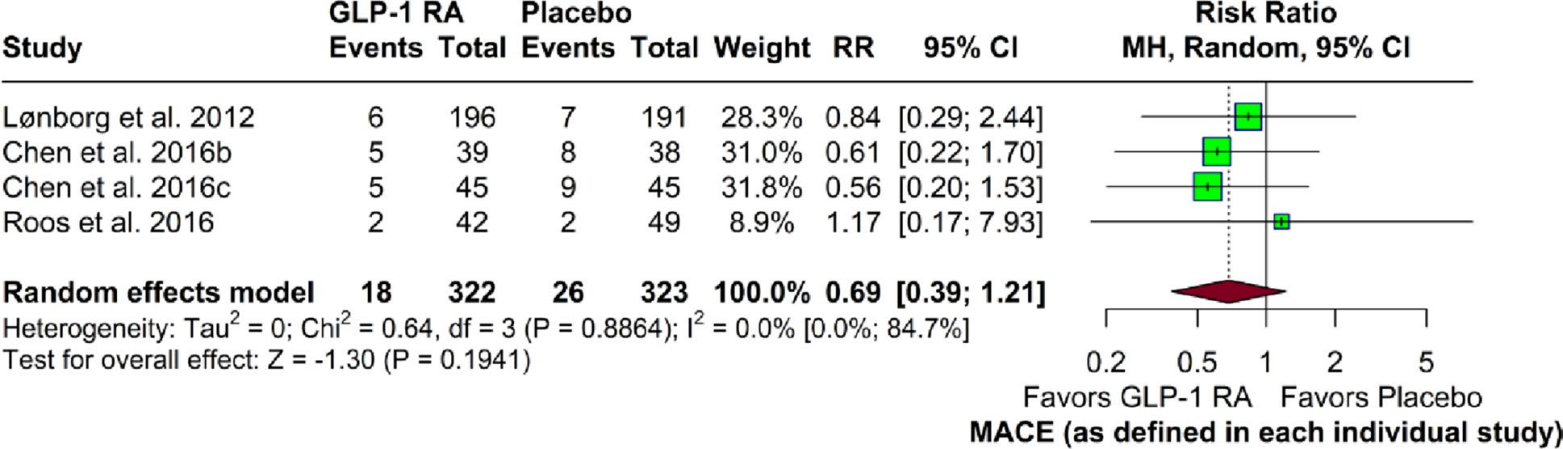
Forest plot of number of patients with a major adverse cardiovascular event (MACE) (as defined in each individual study)

There was a numerically lower risk of major adverse cardiovascular events (MACE) as defined in each study (RR = 0.69; 95% CI: 0.39–1.21; *P* = 0.19; see Fig. 5). However, this difference did not reach statistical significance. This analysis is exploratory and the study-level definitions of MACE are outlined in the supplementary data (see Supplementary Table S3).

Regarding the secondary safety outcomes, no statistically significant difference was detected in hypoglycemia risk (RR = 1.54; 95% CI: 0.79–3.00; *P* = 0.20), although the point estimate suggested a greater risk in the GLP-1 RA group (see Supplementary Figure S3). No cases of pancreatitis were reported in any of the included studies. Further forest plots and sensitivity analyses for these secondary safety outcomes are provided in the supplementary data.

## Discussion

This systematic review and meta-analyses are the largest and most comprehensive evaluations of GLP-1 RAs in AMI to date. The present meta-analyses of RCTs encompass 1,468 participants, which is the greatest number of included patients in any such meta-analyses. Prior meta-analyses were limited by incomplete inclusion of available trials: Previous meta-analyses by Huang et al. [20] did not include the RCT from García Del Blanco et al. with 378 patients [14]. Previous meta-analyses by Yang et al. [22] did not include the RCTs from Lønborg et al. [6], Roos et al. [8], Woo et al. [10], and García Del Blanco [14]. The meta-analyses by Wong et al. [21] did not include the study from Chen et al. [11] with 284 patients. In the present meta-analyses with inclusion of all available RCTs, GLP-1 RAs are associated with a significant reduction in infarct size when indexed to the area at risk, as well as a measurable improvement in LVEF in this population.

The observed reduction in infarct size is clinically meaningful, as infarct size is a key determinant of long-term cardiovascular outcomes such as heart failure and mortality following MI [2]. Notably, the reduction in relative infarct size was consistent across the included randomized trials, with no statistical heterogeneity. Mechanistically, this reduction in infarct size is supported by a substantial amount of preclinical evidence. In-vitro and in-vivo studies in rats and pigs demonstrated that GLP-1 RAs attenuate ischemia–reperfusion injury through multiple ways. These include activation of pro-survival signaling cascades such as PI3K–Akt, reduction of oxidative stress, and suppression of proinflammatory signals after ischemia [28–30].

In addition to their effects on the extent of myocardial injury, GLP-1 RAs were associated with a statistically significant improvement in LVEF. Increases in systolic function following MI of this magnitude may translate into clinically relevant benefits over time. LVEF recovery was associated with a decreased risk of sudden cardiac arrest and mortality in cohort studies [31]. This improvement in LVEF by GLP-1 RAs is likely mediated by both reduced infarct size and additional direct myocardial effects. Previous research has shown that GLP-1 RAs improved endothelial function, enhanced myocardial glucose uptake during myocardial ischemia in rats, and reduced myocardial stunning after an ischemic event in dogs [32–34]. LVEF improvement was observed in the liraglutide subgroup and not with exenatide. This suggests that the beneficial impact might be specific to certain GLP-1 RAs rather than generalizable across all GLP-1 RAs.

The findings suggest that GLP-1 RAs may exert an acute cardioprotective effect on LVEF when administered peri-procedurally during revascularization for AMI. In contrast, improvement in LVEF has not been demonstrated with chronic GLP-1 RA therapy in heart failure patients without AMI [35]. This suggests that the post-MI improvement may be mediated through direct attenuation of ischemia-reperfusion injury. GLP-1 RAs have anti-apoptotic effects through activation of cAMP [36], AMP-activated protein kinase (AMPK)/PI3K-Akt [36] and protein kinase G (PKG)/protein kinase C epsilon (PKCε)/extracellular signal-regulated kinase 1/2 (ERK1/2) pathways [37]. These pathways are maximally relevant in acutely ischemic hearts and ineffective in chronic heart failure with scarred, fibrosed, and already remodeled myocardium [37].

Although there were improvements in surrogate markers, the reduction in major adverse cardiovascular events (MACE) did not reach statistical significance. Several limitations may explain this discrepancy. First, most of the included studies had a relatively small sample size. This limits the statistical power for clinical endpoints such as MACE. Second, the follow-up period (typically 3–6 months) may not have been long enough to detect differences in hard clinical outcomes, as these often require extended follow-up durations. Third, variability in study design, patient characteristics, and definitions of clinical endpoints may have further limited the ability to detect such differences. The variability in study design included differences in the specific GLP-1 RA administered (exenatide vs. liraglutide), dosing regimens, timing of administration (pre-, peri-, or post-reperfusion), and treatment duration.

Overall, the available short-term trial data did not identify any major safety concerns, apart from increased nausea. The most common adverse effect was nausea. It occurred more often with treatment. However, it did not lead to significant study discontinuation. The nausea was mild and manageable in many cases. Importantly, rates of hypoglycemia or pancreatitis were not significantly elevated.

Most included studies enrolled STEMI patients with primary percutaneous coronary intervention. This restricts the generalizability of the findings to those with NSTEMI or non-invasive management. Notably, the primary outcome was demonstrated for STEMI patients only. The LVEF improvement and MACE analyses, however, were based on both STEMI and NSTEMI patients. It remains unclear whether the benefit of LVEF improvement is present equally in both populations. The only included NSTEMI study showed a statistically significant increase in LVEF by a mean difference of 4.6% points [13]. For MACE as defined in the individual study, this same NSTEMI study showed a reduction of the point estimate with a risk ratio of 0.56. However, in the setting of a small sample size of 90 patients, the result was not statistically significant. Further RCTs that include NSTEMI patients are needed. This would allow for more differentiated statements on both populations. This distinction is pathophysiologically relevant, as the nature and severity of ischemia-reperfusion injury differ between STEMI and NSTEMI [38]. A STEMI is typically caused by a complete occlusion of a coronary artery and leads to transmural ischemia. In contrast, NSTEMI is mostly caused by subtotal occlusion of a coronary artery with subendocardial ischemia. For this reason, the ischemic burden, area at risk, and degree of reperfusion injury after revascularization are expected to be smaller in patients with NSTEMI [38]. The cardioprotective effects of GLP-1 RAs are thought to be mediated by an attenuation of ischemia-reperfusion injury. Ischemia-reperfusion injury is mediated by inflammation [39]. Prior experimental studies in mice showed that GLP-1 RAs suppressed proinflammatory signals after myocardial ischemia. The inflammatory response after AMI can lead to adverse left ventricular remodeling, pathologic fibrosis, heart failure, and worse clinical outcomes. Dampening harmful inflammation in order to reduce myocardial injury is an important target in cardiovascular research [30, 40]. The potential underlying processes of the cardioprotective effect of GLP-1 RAs may involve better glucose regulation. However, the early cardiovascular benefits seen in acute settings following AMI indicate that additional mechanisms beyond simple glycemic reduction are involved [41]. It remains to be established whether these benefits translate to the same degree in NSTEMI.

Another limitation of these systematic review and meta-analyses is that no protocol was prospectively registered in a protocol registry.

Overall, these results are consistent with and extend previous experimental and early clinical data. They further support the hypothesis that GLP-1 RAs may have intrinsic cardioprotective properties beyond their known metabolic benefits. However, the absence of proven benefits in major clinical outcomes underscores the need for larger, sufficiently powered trials.

## Conclusions

Among patients experiencing AMI, treatment with GLP-1 RAs reduces relative infarct size and improves LVEF. Overall, no clear safety signal was detected in the available short-term trial data, except for increased nausea. These findings indicate that GLP-1 RAs show promising signals for infarct-size reduction and LVEF improvement, but larger adequately powered RCTs are required before clinical adoption in AMI.

## Supplementary Information

Below is the link to the electronic supplementary material.

Supplementary Material 1.

## Data Availability

No datasets were generated or analysed during the current study.

## Abbreviations

AAR: Area at risk
AMI: Acute myocardial infarction
AMPK: AMP-Activated Protein Kinase BMI: body mass index
CENTRAL: Cochrane Central Register of Controlled Trials
CMR: Cardiovascular magnetic resonance
CI: Confidence interval
Embase: Excerpta Medica dataBASE
ERK1/2: Extracellular signal-regulated kinase 1/2
GLP-1: Glucagon-like peptide-1
GLP-1 RA: Glucagon-like peptide-1 receptor agonist
IV: Intravenous
LVEF: Left ventricular ejection fraction
MACE: Major adverse cardiovascular events
MD: Mean difference
MI: Myocardial infarction
NOS: Newcastle-Ottawa Scale
NSTEMI: Non-ST-elevation myocardial infarction
NR: Not reported
PCI: Percutaneous coronary intervention
PI3K: Phosphatidylinositol-3 kinase
PKCε: Protein kinase C epsilon
PKG: Protein kinase G
PRISMA: Preferred Reporting Items for Systematic Reviews and Meta-Analyses
RCT: Randomized controlled trial
RoB 2: Cochrane Risk of Bias 2
RR: Risk ratio
SC: Subcutaneous
STEMI: ST-elevation myocardial infarction
