# Supplementary material for: A systematic review and meta-analyses of glucagon-like peptide-1 receptor agonists in acute myocardial infarction
Source: Egypt Heart J. 2026 Jul 21;78:55. doi: 10.1186/s43044-026-00767-y (PMC13388624; doi:10.1186/s43044-026-00767-y)
Supplement: Supplementary file 1 — Supplementary Material 1. [file 43044_2026_767_MOESM1_ESM.docx]

**A Systematic Review and Meta-Analyses of Glucagon-Like Peptide-1 Receptor Agonists in Acute Myocardial Infarction**

(Supplementary Material)

# Supplementary data for search strategy

**Supplementary Table S1.** Supplementary data for search strategy

| **Database** | **Search string** |
| --- | --- |
| Medical Literature Analysis and Retrieval System Online (MEDLINE) searched via PubMed.gov | (GLP[Title] OR glucagon-like[Title] OR exenatide[Title] OR albiglutide[Title] OR taspoglutide[Title] OR dulaglutide[Title] OR lixisenatide[Title] OR semaglutide[Title] OR efpeglenatide[Title]) AND (myocardial infarction[Title]) |
| Excerpta Medica dataBASE (Embase) searched via Embase.com (Elsevier) | 'myocardial infarction':ti AND (glp:ti OR 'glucagon like':ti OR exenatide:ti OR albiglutide:ti OR taspoglutide:ti OR dulaglutide:ti OR lixisenatide:ti OR semaglutide:ti OR efpeglenatide:ti) |
| ClinicalTrials.gov | Condition/disease: “Myocardial Infarction” Intervention/treatment: “GLP-1” Study Status: “All studies” |
| Cochrane accessed via Wiley on <https://www.cochranelibrary.com/> | In the Cochrane Library's Advanced Search editor was used - Line #1: GLP OR glucagon-like OR exenatide OR albiglutide OR taspoglutide OR dulaglutide OR lixisenatide OR semaglutide OR efpeglenatide (set the field dropdown to "Title")  - Line #2: "myocardial infarction" → set the field dropdown to "Title"  - Combine the two lines with AND |
| Google Scholar | allintitle: myocardial infarction AND (GLP OR glucagon-like OR exenatide OR albiglutide OR taspoglutide OR dulaglutide OR lixisenatide OR semaglutide OR efpeglenatide) |

# Supplementary data for risk of bias assessment


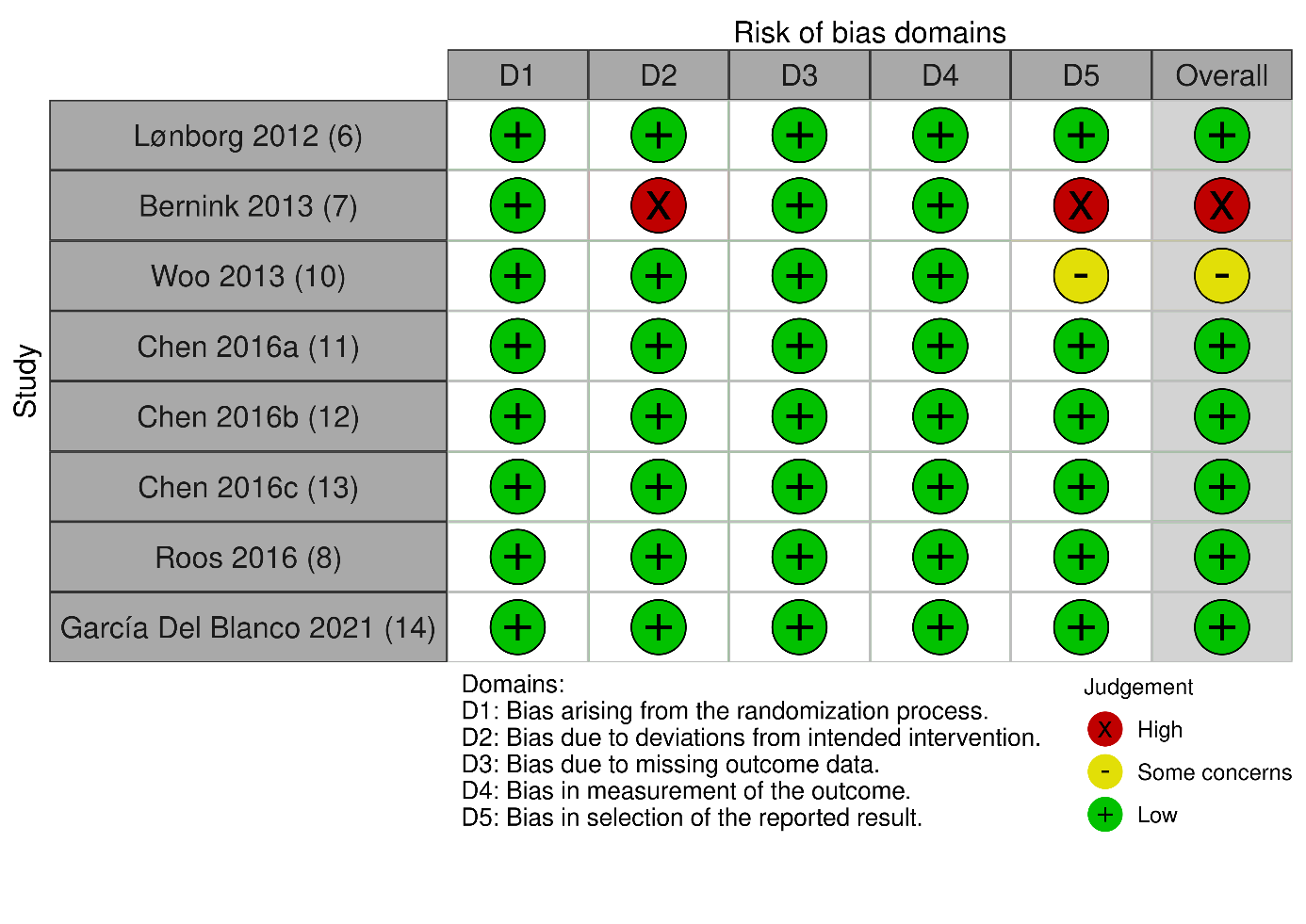


**Supplementary Figure S1.** Risk of bias assessment for included randomized studies. Individual study-level judgments were evaluated using the Cochrane Risk of Bias 2 (RoB 2) tool (24) and are presented as a traffic light plot.


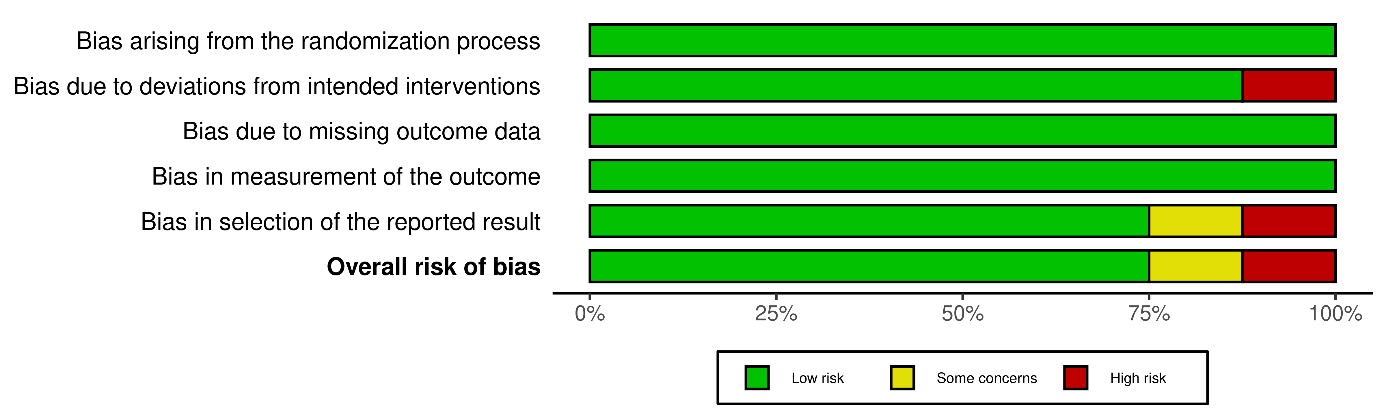


**Supplementary Figure S2.** Risk of bias assessment for included randomized studies. Individual study-level judgments were evaluated using the Cochrane Risk of Bias 2 (RoB 2) tool (24) and are presented as a summary plot.

**Supplementary Table S2.** Risk of bias assessment for included retrospective study, as measured by the Newcastle-Ottawa Scale (NOS) for cohort studies (26)

| **Authors (Year)** | **Study type** | **Selection** | | | | **Comparability** | **Outcome** | | | **Final score** |
| --- | --- | --- | --- | --- | --- | --- | --- | --- | --- | --- |
|  |  | **Representativeness of the Exposed Cohort** | **Selection of the Non-Exposed Cohort** | **Ascertainment of Exposure** | **Demonstration That Outcome of Interest Was Not Present at Start of Study** | **Comparability of Cohorts on the Basis of the Design or Analysis** | **Assessment of Outcome** | **Was Follow-Up Long Enough for Outcomes to Occur** | **Adequacy of Follow Up of Cohorts** |  |
| Nozue et al. (2016) | Cohort | * | * | * | * | ** | * | * | * | 9 |

# Supplementary data for major adverse cardiovascular events (MACE)

**Supplementary Table S3.** Study-level definitions of MACE

| **Study** | **Study-level definition of MACE** |
| --- | --- |
| Lønborg et al. 2012 | Cardiac death, myocardial infarction, stent thrombosis, and stroke at one month |
| Chen et al. 2016b | Repeated revascularization, myocardial infarction, and cardiac death at six months |
| Chen et al. 2016c | Myocardial infarction, repeated revascularization, cardiac death, and stroke at six months |
| Roos et al. 2016 | Cardiac death, myocardial infarction, coronary artery bypass grafting, or repeat percutaneous coronary intervention at four months |

# Supplementary forest plot for the safety outcome of hypoglycemia


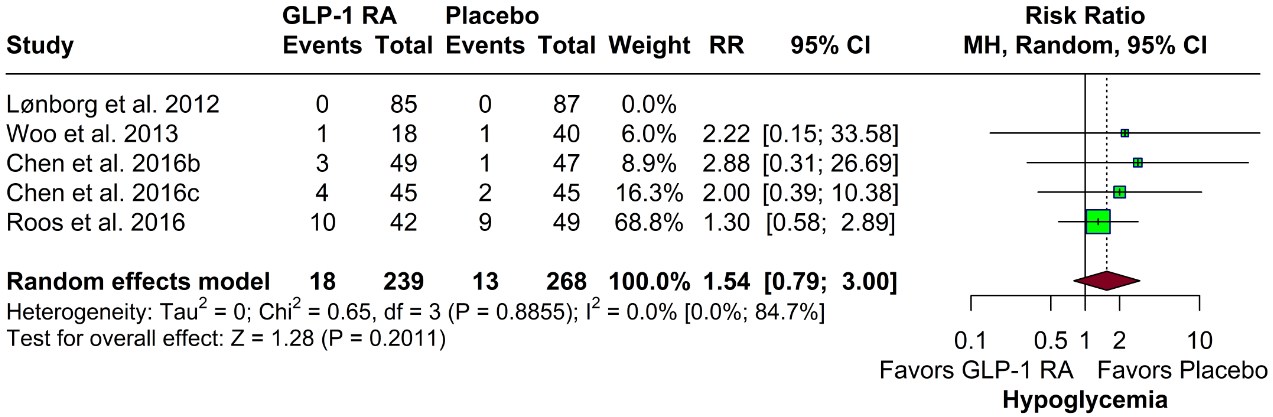
**Supplementary Figure S3.** Forest plot of hypoglycemia

# Supplementary sensitivity analyses

**
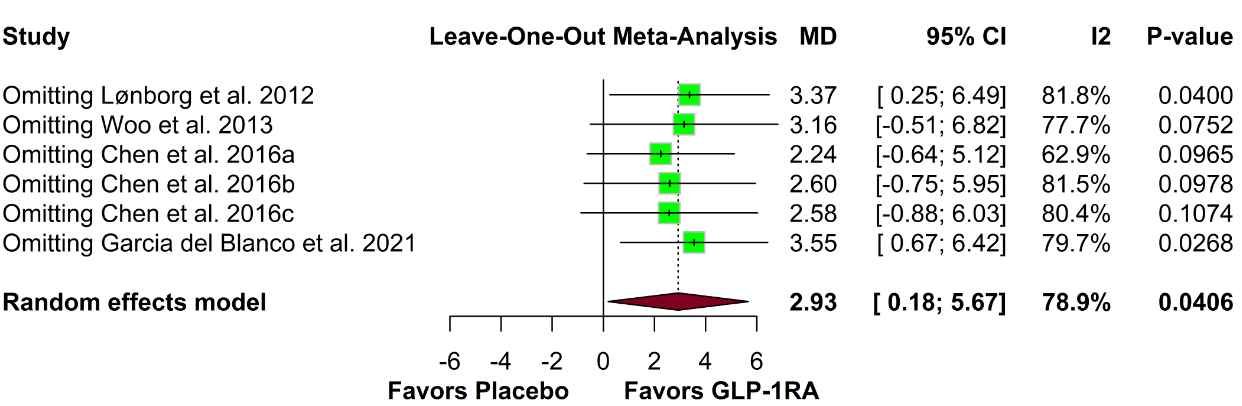
 Supplementary Figure S4.** Leave-one-out sensitivity analysis for left ventricular ejection fraction (LVEF)

**
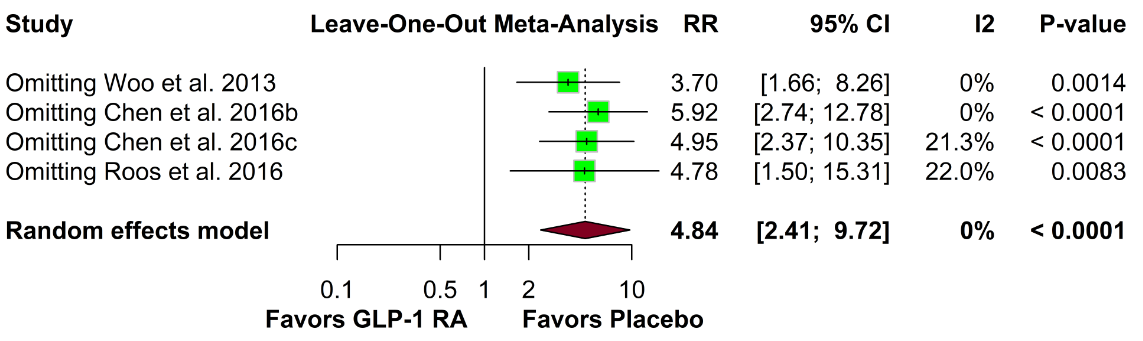
 Supplementary Figure S5.** Leave-one-out sensitivity analysis for nausea

# Supplementary data for PRISMA 2020 for Abstracts checklist

**Supplementary Table S4.** PRISMA 2020 for Abstracts checklist (19)

| **Section and Topic** | **Item #** | **Checklist item** | **Reported (Yes/No)** |
| --- | --- | --- | --- |
| **TITLE** | | |  |
| Title | 1 | Identify the report as a systematic review. | Yes |
| **BACKGROUND** | | |  |
| Objectives | 2 | Provide an explicit statement of the main objective(s) or question(s) the review addresses. | Yes |
| **METHODS** | | |  |
| Eligibility criteria | 3 | Specify the inclusion and exclusion criteria for the review. | Yes |
| Information sources | 4 | Specify the information sources (e.g. databases, registers) used to identify studies and the date when each was last searched. | Yes |
| Risk of bias | 5 | Specify the methods used to assess risk of bias in the included studies. | Yes |
| Synthesis of results | 6 | Specify the methods used to present and synthesise results. | Yes |
| **RESULTS** | | |  |
| Included studies | 7 | Give the total number of included studies and participants and summarise relevant characteristics of studies. | Yes |
| Synthesis of results | 8 | Present results for main outcomes, preferably indicating the number of included studies and participants for each. If meta-analysis was done, report the summary estimate and confidence/credible interval. If comparing groups, indicate the direction of the effect (i.e. which group is favoured). | Yes |
| **DISCUSSION** | | |  |
| Limitations of evidence | 9 | Provide a brief summary of the limitations of the evidence included in the review (e.g. study risk of bias, inconsistency and imprecision). | Yes |
| Interpretation | 10 | Provide a general interpretation of the results and important implications. | Yes |
| **OTHER** | | |  |
| Funding | 11 | Specify the primary source of funding for the review. | N/a |
| Registration | 12 | Provide the register name and registration number. | N/a |

# Supplementary data for PRISMA 2020 Checklist

**Supplementary Table S5.** PRISMA 2020 checklist (19)

| **Section and Topic** | **Item #** | **Checklist item** | **Location where item is reported** |
| --- | --- | --- | --- |
| **TITLE** | | |  |
| Title | 1 | Identify the report as a systematic review. | Title |
| **ABSTRACT** | | |  |
| Abstract | 2 | See the PRISMA 2020 for Abstracts checklist. | “Abstract” section; “PRISMA 2020 for Abstracts checklist” section in supplementary data |
| **INTRODUCTION** | | |  |
| Rationale | 3 | Describe the rationale for the review in the context of existing knowledge. | 3^rd^ paragraph of “Background” section |
| Objectives | 4 | Provide an explicit statement of the objective(s) or question(s) the review addresses. | 4^th^ paragraph of “Background” section |
| **METHODS** | | |  |
| Eligibility criteria | 5 | Specify the inclusion and exclusion criteria for the review and how studies were grouped for the syntheses. | “Study selection” section |
| Information sources | 6 | Specify all databases, registers, websites, organisations, reference lists and other sources searched or consulted to identify studies. Specify the date when each source was last searched or consulted. | “Search strategy” section |
| Search strategy | 7 | Present the full search strategies for all databases, registers and websites, including any filters and limits used. | “Search strategy”; Supplementary Table S1 section |
| Selection process | 8 | Specify the methods used to decide whether a study met the inclusion criteria of the review, including how many reviewers screened each record and each report retrieved, whether they worked independently, and if applicable, details of automation tools used in the process. | “Study selection” section |
| Data collection process | 9 | Specify the methods used to collect data from reports, including how many reviewers collected data from each report, whether they worked independently, any processes for obtaining or confirming data from study investigators, and if applicable, details of automation tools used in the process. | 1^st^ and 2^nd^ paragraph of “Outcome measures” section |
| Data items | 10a | List and define all outcomes for which data were sought. Specify whether all results that were compatible with each outcome domain in each study were sought (e.g. for all measures, time points, analyses), and if not, the methods used to decide which results to collect. | 1st and 2nd paragraph of “Outcome measures” section; Supplementary Table S3 section |
|  | 10b | List and define all other variables for which data were sought (e.g. participant and intervention characteristics, funding sources). Describe any assumptions made about any missing or unclear information. | 2^nd^ paragraph of “Outcome measures” section and Tables 1-2 |
| Study risk of bias assessment | 11 | Specify the methods used to assess risk of bias in the included studies, including details of the tool(s) used, how many reviewers assessed each study and whether they worked independently, and if applicable, details of automation tools used in the process. | “Risk of bias assessment” section |
| Effect measures | 12 | Specify for each outcome the effect measure(s) (e.g. risk ratio, mean difference) used in the synthesis or presentation of results. | “Statistical methods” section |
| Synthesis methods | 13a | Describe the processes used to decide which studies were eligible for each synthesis (e.g. tabulating the study intervention characteristics and comparing against the planned groups for each synthesis (item #5)). | 1^st^ paragraph of “Study selection” section |
|  | 13b | Describe any methods required to prepare the data for presentation or synthesis, such as handling of missing summary statistics, or data conversions. | 2^nd^ paragraph of “Outcome measures” section |
|  | 13c | Describe any methods used to tabulate or visually display results of individual studies and syntheses. | “Quality assessmen” section; “Risk of bias assessment” section; Figure captions underneath Figure 1-5; Supplementary Figures S1-S5 |
|  | 13d | Describe any methods used to synthesize results and provide a rationale for the choice(s). If meta-analysis was performed, describe the model(s), method(s) to identify the presence and extent of statistical heterogeneity, and software package(s) used. | 1^st^-3^rd^ paragraph of “Statistical methods” section |
|  | 13e | Describe any methods used to explore possible causes of heterogeneity among study results (e.g. subgroup analysis, meta-regression). | 2^nd^-3^rd^ parapgraph of “Statisical methods” section |
|  | 13f | Describe any sensitivity analyses conducted to assess robustness of the synthesized results. | 2^nd^ parapgraph of “Statisical methods” section |
| Reporting bias assessment | 14 | Describe any methods used to assess risk of bias due to missing results in a synthesis (arising from reporting biases). | “Risk of bias assessment” section; “Supplementary data for risk of bias assessment“ section in supplemenary data |
| Certainty assessment | 15 | Describe any methods used to assess certainty (or confidence) in the body of evidence for an outcome. | GRADE approach is not reported in this publication |
| **RESULTS** | | |  |
| Study selection | 16a | Describe the results of the search and selection process, from the number of records identified in the search to the number of studies included in the review, ideally using a flow diagram. | “Flow diagram of the study search selection process” section |
|  | 16b | Cite studies that might appear to meet the inclusion criteria, but which were excluded, and explain why they were excluded. | not reported |
| Study characteristics | 17 | Cite each included study and present its characteristics. | Table 1 and Table 2 in “Characteristics of included studies” section |
| Risk of bias in studies | 18 | Present assessments of risk of bias for each included study. | “Supplementary data for risk of bias” section in supplementary data |
| Results of individual studies | 19 | For all outcomes, present, for each study: (a) summary statistics for each group (where appropriate) and (b) an effect estimate and its precision (e.g. confidence/credible interval), ideally using structured tables or plots. | “Primary outcome: myocardial infarction size in grams in relation to the area at risk (AAR) in grams” section; All paragraphs of “Secondary outcomes” section |
| Results of syntheses | 20a | For each synthesis, briefly summarise the characteristics and risk of bias among contributing studies. | not reported |
|  | 20b | Present results of all statistical syntheses conducted. If meta-analysis was done, present for each the summary estimate and its precision (e.g. confidence/credible interval) and measures of statistical heterogeneity. If comparing groups, describe the direction of the effect. | “Primary outcome: myocardial infarction size in grams in relation to the area at risk (AAR) in grams” section; All paragraphs of “Secondary outcomes” section |
|  | 20c | Present results of all investigations of possible causes of heterogeneity among study results. | “Primary outcome: myocardial infarction size in grams in relation to the area at risk (AAR) in grams” section; 1^st^ paragraph of “Left ventricular ejection fraction as a significant secondary outcome” section, and 5^th^ paragraph of section “Discussion” |
|  | 20d | Present results of all sensitivity analyses conducted to assess the robustness of the synthesized results. | “Primary outcome: myocardial infarction size in grams in relation to the area at risk (AAR) in grams” section; 2^nd^ paragraph of “Left ventricular ejection fraction as a significant secondary outcome”, section; 3^rd^ paragraph of “Other secondary outcome” section; Supplementary Figure S4 section and Supplementary Figure S5 |
| Reporting biases | 21 | Present assessments of risk of bias due to missing results (arising from reporting biases) for each synthesis assessed. | not reported |
| Certainty of evidence | 22 | Present assessments of certainty (or confidence) in the body of evidence for each outcome assessed. | GRADE approach is not reported in this publication |
| **DISCUSSION** | | |  |
| Discussion | 23a | Provide a general interpretation of the results in the context of other evidence. | Paragraphs 1, 2, 3, 4, 5, 6, 7, and 9 of the “Discussion” section |
|  | 23b | Discuss any limitations of the evidence included in the review. | Paragraphs 5, 6, 7, and 9 of the “Discussion” section |
|  | 23c | Discuss any limitations of the review processes used. | Paragraphs 8 of the “Discussion” section |
|  | 23d | Discuss implications of the results for practice, policy, and future research. | Paragraphs 7 and 9 of the “Discussion” section and “Conclusions” section |
| **OTHER INFORMATION** | | |  |
| Registration and protocol | 24a | Provide registration information for the review, including register name and registration number, or state that the review was not registered. | n/a |
|  | 24b | Indicate where the review protocol can be accessed, or state that a protocol was not prepared. | n/a |
|  | 24c | Describe and explain any amendments to information provided at registration or in the protocol. | n/a |
| Support | 25 | Describe sources of financial or non-financial support for the review, and the role of the funders or sponsors in the review. | “Funding” section |
| Competing interests | 26 | Declare any competing interests of review authors. | n/a |
| Availability of data, code and other materials | 27 | Report which of the following are publicly available and where they can be found: template data collection forms; data extracted from included studies; data used for all analyses; analytic code; any other materials used in the review. | “References” section |

# Supplementary references

24. Sterne JAC, Savović J, Page MJ, Elbers RG, Blencowe NS, Boutron I, et al. RoB 2: a revised tool for assessing risk of bias in randomised trials. BMJ. 2019;366:l4898.

26. Wells GA SB, O'Connell D, Peterson J, Welch V, Losos M, Tugwell P. The Newcastle-Ottawa Scale (NOS) for assessing the quality of nonrandomised studies in meta-analyses: The Ottawa Hospital Research Institute; [cited 2026 April 30, at 15:01]. Available from: https://ohri.ca/en/who-we-are/core-facilities-and-platforms/ottawa-methods-centre/newcastle-ottawa-scale.

19. Page MJ, McKenzie JE, Bossuyt PM, Boutron I, Hoffmann TC, Mulrow CD, et al. The PRISMA 2020 statement: an updated guideline for reporting systematic reviews. Bmj. 2021;372:n71.
